# Factors contributing to the persistence of undernutrition among children under 5 years of age in Rwanda: a community participatory qualitative study

**DOI:** 10.1080/16549716.2025.2581454

**Published:** 2025-11-26

**Authors:** Madeleine Mukeshimana, Theoneste Ntakirutimana, Marie Laetitia Ishimwe Bazakare, Gunilla Krantz

**Affiliations:** a School of Nursing and Midwifery, University of Rwanda, Kigali, Rwanda; b School of Public Health, University of Rwanda, Kigali, Rwanda; c Monitoring, Evaluation, Research and Learning Department, Rwanda Society of Obstetricians and Gynecologists, Kigali, Rwanda; d School of Public Health and Community Medicine, Institute of Medicine, University of Gothenburg, Gothenburg, Sweden

**Keywords:** undernutrition, children, <5 years, participatory research, open-space meeting, thematic analysis

## Abstract

**Background:**

Undernutrition remains a significant global health challenge, particularly among children <5 years of age. Despite efforts to combat this issue, millions of children worldwide, especially in low-income countries such as Rwanda, continue to suffer from undernutrition, which adversely affects their health, growth, and development.

**Objective:**

This study aimed to explore and analyse the factors contributing to the persistence of undernutrition among children <5 years of age in Rwanda that are not adequately addressed by existing community interventions.

**Methods:**

A participatory research design with the open-space meeting method was used to collect the primary data. Eight open-space meetings were conducted in two selected districts in the Northern Province of Rwanda. A total of 194 participants, including community health workers, parents/caregivers, and healthcare professionals, participated in discussions. Thematic analysis using Atlas.ti software was used to analyse the qualitative data.

**Results:**

The findings revealed a complex interplay of factors contributing to the persistence of undernutrition among children <5 years of age in Rwanda. Key factors identified included conflicts among parents, limited involvement of male parents in childcare, the mental health status of children, inexperienced home caregivers, and inadequate family planning practices.

**Conclusion:**

Despite concerted efforts by governmental and non-governmental organizations, undernutrition among children <5 years of age remains a pressing issue in Rwanda. This study highlights specific household-related factors not adequately targeted by existing interventions and proposes potential strategies to address them effectively, aiming to reduce undernutrition among children <5 years in Rwanda.

## PAPER CONTEXT

● **Main findings:** Household-related social and behavioural factors, such as parental conflict, are key contributors to persistent undernutrition among children <5 years of age in Rwanda.

● **Added knowledge:** The study highlights under-addressed psychosocial and caregiving factors contributing to persistent undernutrition among children <5 years in Rwanda.

● **Global health impact on policy and action:** The findings support the need for integrated family-centred nutrition interventions that include psychosocial support, male engagement, and caregiver education to improve child nutrition outcomes globally.

## Background

Globally, ~45.4 million deaths among children <5 years of age are attributed to undernutrition, with a disproportionate burden in low-income countries [1]. Despite progress in recent years, millions of children worldwide still suffer from undernutrition, which poses serious threats to their health, growth, and development [1].

Undernutrition manifests in various forms, such as stunting (low height-for-age), wasting (low weight-for-height), and underweight (low weight-for-age), and is often exacerbated by factors such as inadequate dietary intake, poor feeding practices, limited access to healthcare, and socioeconomic disparities. In low- and middle-income countries, undernutrition remains persistent, affecting approximately 149 million children <5 years of age; the highest burden occurs in sub-Saharan Africa and South Asia [2].

Undernutrition not only has physical consequences but also leads to psychological, economic, and social impacts. Beyond physical health, undernutrition contributes to cognitive impairment, a weakened immune system, and increased susceptibility to disease [3]. Undernourished children are more likely to experience long-term consequences such as poor academic performance, reduced productivity, and perpetuation of intergenerational cycles of poverty [3].

The Rwanda Demographic and Health Survey conducted in 2019 revealed that stunting, on average, affected 32% of Rwandan children <5 years of age; 41% of children were stunted in the Northern Province, and wasting and underweight affected 2% and 6% of children, respectively [4].

Several factors contribute to undernutrition in Rwanda, including limited access to nutritious food, inadequate healthcare infrastructure, poor healthcare seeking, poor sanitation and hygiene practices, and socioeconomic disparities [5]. Rural-urban disparities exacerbate these challenges; rural children are disproportionately affected by poverty, poorly educated parents, limited access to healthcare services, poor social networks and parental violence [5].

Efforts to address undernutrition in Rwanda are ongoing. The government is implementing various interventions aimed at improving nutritional outcomes. These include promotion of exclusive breastfeeding, micronutrient supplementation, community-based nutrition programmes, and agricultural initiatives to enhance food security [6]. In addition, partnerships with international organizations and local non-governmental organizations have supported initiatives to strengthen healthcare systems, improve access to clean water and sanitation, and enhance nutritional education and awareness among communities. Despite these efforts, significant gaps persist in comprehensively addressing undernutrition in Rwanda, hindered by challenges such as limited resources, infrastructure constraints, and sociocultural factors.

This study explores community-identified factors contributing to the persistence of undernutrition among children <5 years in Gicumbi and Musanze Districts, Northern Province of Rwanda.

## Methods

### Aim of the study

The aim of this study was to explore the factors contributing to the persistence of undernutrition among children <5 years of age in Rwanda, despite all the community interventions that have been implemented to prevent this health issue.

### Research team and roles

The research team comprised the principal investigator (first author), two research assistants, and the co-authors who served as quality controllers. The first author led the data collection process by facilitating all open-space meetings, ensuring that discussions stayed focused and that all participants were actively engaged. Two research assistants from the School of Nursing and Midwifery supported the process by taking detailed field notes, recording the discussions, and transcribing the audio recordings verbatim. They also contributed to preliminary data analysis using Atlas.ti software. The co-authors acted as quality controllers, reviewing the transcripts, verifying the consistency and credibility of the coding process, and providing feedback to ensure the trustworthiness and rigour of the data analysis.

### Study design and approach

This qualitative study used a participatory research design and the open-space meeting method. Participatory research involves active involvement of stakeholders, including community members, throughout the research process [7]. It aims to empower communities, enhance the relevance and applicability of research outcomes, and promote social change.

### Study sites, population and sampling strategy

The target population for this study included community health workers, parents/caregivers of children <5 years, and healthcare professionals working in nutrition services at health centres within the two selected districts in Northern Rwanda.

Inclusion criteria were:

- Community health workers actively serving in the study sites;
- Parents or caregivers of children <5 years who had been living in the study areas for a reasonable period and were knowledgeable about local child nutrition practices;
- Healthcare professionals directly involved in nutrition services at health centres (e.g. nurses in charge of nutrition).

Exclusion criteria were:

- Parents/caregivers without children <5 years;
- Healthcare workers not directly engaged in nutrition-related services.

Eight open-space meetings (each with 20–25 participants) were conducted in two districts of the Northern Province of Rwanda, selected because of their high prevalence of undernutrition among children <5 years [8]. The target sample was 200 participants and 194 were reached, including 109 community health workers, 80 parents/caregivers of children <5 years, and five healthcare professionals (nutrition focal nurses at health centres). Eligible participants were identified with the support of the local community administration, prioritizing those residing in the study sites and those with knowledge of community nutrition practices.

### Data collection procedures

Community leaders were consulted for approval and support before data collection. Meetings were held in schools or office rooms conducive to interactive discussion. Each meeting began with an opening circle where the facilitator introduced the topic: ‘What are the factors contributing to the persistence of undernutrition among children <5 years of age in your community, despite all existing interventions aimed at reducing this issue/problem?’

Participants were then free to propose subtopics and move between discussions using the Law of Two Feet principle. Subtopics that frequently emerged included (a) the role of family dynamics and parental conflict, resulting in intimate partner violence (IPV), (b) male involvement in child-rearing and nutrition, (c) the impact of the mental health of caregivers and children, (d) challenges faced by inexperienced home caregivers, and (e) the influence of family planning practices on child nutrition. All discussions were conducted in Kinyarwanda, the local language, to facilitate open communication and ensure participant comfort. Meetings were audio recorded in Kinyarwanda, and detailed field notes were taken. Discussions lasted approximately 1 hour and ended with a closing circle to summarize the key points.

To ensure linguistic fidelity, audio recordings were transcribed verbatim in Kinyarwanda and then translated into English by a bilingual researcher fluent in both languages. A second bilingual team member reviewed the translations for accuracy. Any discrepancies were discussed and resolved by consensus to ensure the original meaning and context were preserved.

### Data analysis

An inductive, data-driven thematic analysis approach was used to analyse the qualitative data, following Braun and Clarke’s six-step framework [9]. This approach allowed themes to emerge organically from the data without applying a pre-existing theoretical model. Atlas.ti software was used to facilitate coding and organizing the data. A completed COREQ checklist documenting the reporting criteria is included in Appendix 1.

1. **Familiarization**: The research team read and re-read the interview and focus group transcripts to gain a deep understanding of participants’ perspectives.
2. **Generating initial codes**: Significant patterns, phrases, and ideas were identified in the data. These were highlighted and assigned initial codes that captured recurring concepts.
3. **Searching for themes**: Related codes were grouped into broader themes representing key factors contributing to the persistence of undernutrition.
4. **Reviewing themes**: Themes were reviewed and refined to ensure that they accurately represented the data. Some themes were merged or discarded if not sufficiently supported.
5. **Defining and naming themes**: Each final theme was clearly defined and given a descriptive name that captured its essence.
6. **Producing the report**: Themes and subthemes were used to structure the findings, with quotes from participants to illustrate each point.

In addition to transcript analysis, field notes were also reviewed and analysed to complement and enrich transcript data, particularly in interpreting non-verbal cues and the contextual dynamics of the discussions (Table 1).

**Table 1.** Summary of emerged themes and subthemes related to factors contributing to the persistence of undernutrition among children <5 years in Rwanda.

Themes	Definition of theme	Subthemes	Brief description
Inadequate childcare practices	Refers to insufficient or inappropriate daily care routines, poor feeding practices, and lack of parental or caregiver support that directly affect children’s nutritional status	Non-involvement of male parents in the care of children <5 years	Male parents often disengage from childcare, especially with regard to nutrition-related decisions, due to patriarchal norms
		Inexperienced caregivers	Children <5 years are often left with untrained domestic workers (house girls) or young siblings, resulting in poor feeding and care
Compromised mental and emotional well-being of children	Reflects psychosocial and emotional stressors that interfere with children’s appetite, feeding behaviour, and overall development	Maternal neglect	Mothers, often breadwinners, are absent during the day, leaving children without emotional care, affecting feeding
		Intimate partner violence	Exposure to family conflict and violence disrupts children’s emotional security, leading to eating disturbances and anxiety
		Child abuse	Children are sometimes mistreated by domestic workers due to strained employer relationships or lack of supervision
Unmet family planning and nutritional needs	Encompasses structural and cultural issues related to large family size and the resulting inability to provide adequate nutrition for each child	Many children in family	Poor family planning contributes to high child-to-parent ratios, straining resources and limiting attention
		Inadequate nutritional resources	Larger families struggle to consistently provide nutritious food, especially in rural and low-income settings

### Ethical principles

Ethical clearance was obtained from the College of Medicine Institutional Review Board (no. 032/CMHS IRB/2023), and permission from the district mayors of the study settings was received. After obtaining permissions from the study sites, community leaders were contacted to provide support in the recruitment of participants. The participants were given comprehensive information regarding the study and its importance. Signed informed consent was obtained from each participant during data collection. Furthermore, participants were informed that participation was voluntary, that they could withdraw at any time, and that codes were used instead of names for identification. We also kept the recorded data in a safe, locked space at the University of Rwanda. Each participant was reimbursed for transport fees.

## Results

### Sociodemographic characteristics of the participants

A total of 194 participants (132 women, aged 28–48 years; 62 men, aged 35–50 years) from two districts located in the Northern Province of Rwanda participated in our open-space meetings. The 194 participants consisted of 109 community health workers (aged 38–50 years), 80 parents/caregivers of children <5 years, and five healthcare professionals (aged 28–48 years, nurses in charge of nutrition at the health centre).

### Factors contributing to the persistence of undernutrition among children <5 years in Rwanda

Three major themes emerged from the analysis, each representing distinct but interrelated factors contributing to the persistence of undernutrition among children <5 years in Rwanda. A summary of the themes and related subthemes, definitions of the themes and brief descriptions of the subthemes is presented in Table 1. Each theme represents a broad factor influencing undernutrition among children <5 years in Rwanda, and the subthemes provide specific, context-based explanations and examples illustrating how these factors manifest in everyday caregiving environments.

#### Inadequate childcare practices

##### Non-involvement of male parents in the care of children < 5 years

The most cited factor was the lack of male participation in child nutrition and care. Despite holding authority in household decisions, many fathers remain uninvolved in matters of child feeding, leaving mothers to cope alone, often without resources.

My husband does not care if our small child needs this or that … even when I ask him, he just ignores me. I am obliged to feed my baby whatever I have, even just sweet potatoes. It is sad. (female parent, 44years)

Some fathers were reported to misuse family food resources, such as selling milk or eggs meant for the child to buy alcohol, highlighting the urgency of male engagement in child nutrition interventions.

Participants proposed community dialogues led by local leaders, male champion initiatives, and parenting education forums to shift cultural norms and increase male involvement in childcare and nutrition.

##### Inexperienced caregivers

Many families rely on house girls (a local term referring to young, often untrained, female domestic workers) or older siblings for childcare. These caregivers often lack the knowledge or commitment required to ensure proper feeding routines.

The main problem is not a lack of food in the house; it’s that the house girl feeds herself instead of feeding the children. (community health worker, 40years)

Young siblings, when assigned caregiving duties, often neglect feeding schedules due to inexperience or distractions, contributing to persistent undernutrition.

Participants recommended training programmes for household caregivers, and establishment of affordable community-based day care centres managed by trained staff to reduce reliance on unskilled caregivers.

#### Compromised mental and emotional well-being of children

##### Maternal neglect

Mothers returning to work shortly after childbirth shared concerns about reduced breastfeeding and diminished emotional connection with the child:

Upon returning to work, my child’s appetite declined. He refused to breastfeed in the evening, and I think he misses me during the day. (female parent, 38years)

Healthcare workers and parents highlighted how emotional detachment from maternal figures can lead to refusal to eat, poor weight gain, and stunted development.

Participants highlighted the need for flexible work arrangements and community-based psychosocial support groups for working mothers.

##### Intimate partner violence

Participants noted that IPV against women in the household affects not just the victims but also children being exposed to conflict; IPV creates fear, instability, and mental distress that can disrupt children’s eating behaviour and development.

Fights between parents make children scared; they refuse to eat and just cry. (female parent, 42years)

When I’m beaten or abused, I have no energy left to care for my children properly. (female parent, 36years)

Participants suggested that health centres should integrate IPV screening and counselling into maternal and child health services, and community health workers should be trained to support safe reporting.

##### Child abuse

Participants shared distressing accounts of abuse by domestic workers. Poor employer–employee relationships may lead to retaliation, including food deprivation and physical abuse of children.

My baby was subjected to unspeakable cruelty by the house help. She would leave him submerged in water for hours. (female parent, 36 years)

Some housemaids beat our children or withhold food until we return. Mothers need to be careful about who they select as a caregiver. (female parent, 40 years)

Proposed solutions included closer community monitoring of domestic workers, parental vigilance, and basic training and regulation of caregivers. In addition, the establishment of community-based childcare centres, where trained mothers could provide care and receive modest compensation from parents or guardians, was recommended.

#### Unmet family planning and nutritional needs

##### Many children in the family

Despite national efforts promoting family planning, many families still have large numbers of children, often due to cultural or religious beliefs. This reduces the quality and quantity of care and food provided to each child.

Feeding more than five children in one family is almost impossible. (Female parent, 48years)

Participants also cited fear of side effects of contraceptives or opposition from partners as reasons for not using family planning.

Participants recommended increased door-to-door family planning education by community health workers and greater involvement of men and religious leaders to counter myths and resistance.

##### Inadequate nutritional resources

In low-income or rural settings, families often struggle to provide sufficient nutritious food, particularly when there is more than one child <5 years.

Having two small children, both needing eggs, milk … it’s impossible in our low-income household. We have to prioritize or skip days. (male parent, 44 years)

These findings underscore the prevalence of undernutrition within families with multiple children <5 years, particularly among the poorest households, characterized by limited income and resources.

Participants emphasized expanding kitchen gardens, strengthening Girinka (one cow per poor family), and prioritizing households with children <5 years in nutrition-sensitive support programmes.

## Discussion

This study revealed key social, behavioural, and systemic factors that contribute to the persistence of undernutrition among children <5 years in Rwanda, despite national interventions. Beyond identifying the causes, the findings highlight strategic entry points for targeted policy, community, and healthcare-based interventions.

### Non-involvement of male parents in childcare

The lack of paternal involvement, influenced by entrenched patriarchal norms, undermines childcare and nutritional decision-making at the household level [10]. Furthermore, several studies have shown that the presence of mothers and fathers is crucial for children’s growth [11–13]. In Rwanda, the non-involvement of male parents in the care of children is heavily influenced by patriarchal culture. A male parent is seen as an authority figure who does not need to lower himself to shoulder women’s roles, including caring for their children [10,14,15]. Previous studies highlight the father’s influence on resource allocation, but our findings emphasize the urgent need for community-based behavioural change interventions targeting men. Programmes implemented by the Ministry of Gender and Family Promotion and local leaders could integrate culturally adapted community dialogues, male champion initiatives, and parental education forums to shift gender norms and increase male participation in childcare and nutrition.

### Inexperienced caregivers

Our results show that many children <5 years are left in the care of young siblings or untrained domestic workers, leading to improper feeding and neglect. This may lead to adverse effects on their mental health, characterized by a lack of love and care, contributing to anxiety and depression. This, in turn, negatively affects children’s nutritional status; they may refuse to eat or suffer from reduced food intake. Various studies have explained how anxiety and depression in children can lead to malnutrition through disrupted appetite and eating habits, resulting in inadequate intake of the nutrients needed for physical and cognitive development [16]. Moreover, prolonged stress can alter metabolism, leading to unintended weight loss or nutrient deficiencies [17].

In addition, caregivers who lack the necessary knowledge and/or experience may fail to provide proper feeding practices, hygiene, and healthcare for children; they may not know the importance of the appropriate timing for introducing complementary foods; or how to prepare nutritionally balanced meals, which can lead to poor dietary intake and increase the risk of infections, both of which contribute to undernutrition [18,19]. Addressing this requires institutional and structural responses, including the development of community-based childcare centres (daycare) staffed by trained early childhood professionals. The Ministry of Education and the Ministry of Gender and Family Promotion can collaborate with local governments to pilot public daycare programmes in both rural and urban communities. In addition, awareness campaigns on the risks of untrained caregiving could be integrated into routine maternal and child health services.

### Maternal neglect and work demands

Working mothers often face competing demands between income generation and childcare, leading to emotional neglect and undernutrition. This is supported by studies confirming that neglected children often do not receive timely meals, proper medical attention, or nurturing environments that support their healthy development. This neglect exacerbates the risk of undernutrition because children are not given the necessary attention to ensure that their nutritional needs are met [20,21]. We recommend promoting flexible work policies for mothers, particularly in sectors employing large numbers of women. Employers, particularly in the informal sector, should be encouraged through policy incentives to support breastfeeding-friendly environments, on-site crèches, or staggered work hours. Community-based support groups could also provide psychosocial support for working mothers.

### Intimate partner violence

Intimate partner violence emerged as a major barrier to optimal maternal caregiving and infant feeding. Research indicates that women who experience IPV before or during pregnancy are at increased risk of having malnourished children [22–24]. Intimate partner violence can have a detrimental effect on the mental health and psychosocial well-being of maternal caregivers, disrupting crucial bonding between mothers and children and thus influencing maternal feeding practices [25]. According to UNICEF, women experiencing IPV are less likely to engage in recommended breastfeeding practices [26]. They are also more inclined to use alternative formulas, delaying essential breastfeeding, which is critical for the development of a newborn’s immune system, due to the unsupportive environment created by violence [27].

This finding underscores the need to mainstream IPV screening and psychosocial counselling into maternal and child health programmes. Health centres should have the capacity to detect IPV and refer affected mothers for supportive services. Community health workers can be trained to facilitate safe reporting and access to services. Social protection measures must also address IPV as a health and nutrition concern, not just a legal or gender issue.

### Child abuse by caregivers

Child abuse by domestic workers, often due to poor working conditions or lack of oversight, was also found to contribute to undernutrition. This finding is supported by the results of several studies. Morales et al. [20] stated that child neglect, a common form of child abuse, can lead to inadequate food provision and poor feeding practices, which directly affect a child’s nutritional status [20]. Neglected children often experience prolonged hunger, improper diets, and a lack of the essential nutrients necessary for growth and development [20,21]. Carpenter et al. [28] also reported that emotional abuse and chronic stress have a direct impact on children’s physical health; high levels of stress hormones such as cortisol can impair growth, appetite, and digestion, further worsening malnutrition.

In addition, abused children tend to have a higher risk of recurrent illnesses due to a weak immune system, compounding the problem; they often lack timely medical care and support, making recovery from infections and associated malnutrition more difficult [20,21].

The prevalence of such abuse in Rwanda is alarming, with reported cases of child fatalities and severe maltreatment at the hands of caregivers. Despite this, community interventions to combat undernutrition often overlook critical issues. Strategies to curb this issue should include regulation and training of domestic workers, parental education on non-violent disciplinary methods, and community vigilance systems. The Rwanda National Child Development Agency could collaborate with local administrative leaders to enforce basic standards in domestic caregiving and raise awareness about signs of child abuse.

### Poor family planning

Our findings reinforce the existing literature linking high child-to-parent ratios and undernutrition. Mookerjee et al. [29] underscored the positive impact of family planning on child health, indicating that contraception led to improvements in children’s height-for-age and reduced stunting. Bigirimana [30] found a significant association between unmet family planning needs and stunting among Rwandan children. Family planning plays a crucial role in reducing undernutrition among children <5 years [31,32] because it enables birth spacing and provides parents with time to care for their children and address their needs. In Rwanda, the uptake of family planning has increased, but challenges remain, including myths about contraceptives, such as the belief that some family planning methods cause different cancers in women.

Addressing this requires strengthened family planning services, especially in rural and hard-to-reach areas. Efforts must go beyond service provision to tackle myths, misinformation, and male opposition to contraceptive use. Community health workers and peer educators should be mobilized to provide door-to-door education, and religious and cultural leaders can be advocates for responsible parenthood.

### Inadequate nutritional resources

Limited access to diverse, affordable, and nutritious food remains a barrier, especially in larger families. This issue of inadequate nutritional resources is common in different low-income countries and is one of the risk factors for undernutrition among children, as confirmed by different studies [33,34]. Although Rwanda has implemented agriculture-based nutrition interventions, such as kitchen gardens and Girinka (one cow per poor family) [35,36], our findings suggest a need to scale up and better monitor these programmes. Households with children <5 years should be prioritized in agricultural distribution, home fortification programmes, and social protection schemes such as nutrition-sensitive cash transfers. Local nutrition monitoring committees can help track and address acute food insecurity.

Across all themes, participants proposed practical, context-sensitive strategies to address undernutrition, from engaging men and strengthening childcare to scaling nutrition-sensitive agriculture. These community-driven solutions are supported by existing evidence [10,37–39] and provide clear entry points for policy and action in Rwanda.

### Study limitations

This study has some limitations. First, data were collected in only two districts, which may limit the the transferability of the findings to other settings. Second, the open-space approach depended on participants’ self-reported perceptions, which could be influenced by social desirability bias. Third, although the method promoted broad and inclusive participation, it limited the depth of exploration of individual experiences. Nevertheless, the inclusion of diverse participant groups and the participatory nature of the open-space meetings generated rich, contextually grounded insights that enhance the study’s credibility.

## Conclusion

This community-based qualitative study provides critical insights into the persistent problem of undernutrition among children <5 years in Rwanda. Despite national and local efforts to address this issue, our findings highlight several social and structural factors that continue to hinder progress. These include the non-involvement of male parents in childcare, reliance on inexperienced caregivers, maternal neglect, IPV, child abuse, limited family planning, and inadequate nutritional resources.

Participants not only described these challenges but also proposed practical solutions, such as increasing male engagement in childcare, training caregivers, expanding access to daycare services, integrating IPV screening into health programmes, strengthening family planning education, and scaling up nutrition-sensitive support. Their recommendations reinforce the value of participatory approaches in shaping responsive, family-centred interventions.

A multisectoral strategy that integrates these community-driven insights is essential to reduce undernutrition and improve child well-being in Rwanda.

## Supplementary Material

Appendix 7Completed COREQ checklist M.docx

## Data Availability

The datasets used and/or analysed in this study are available from the corresponding author upon request.

## Appendix. Completed COREQ checklist:

‘*Factors contributing to the persistence of undernutrition among under five children in Rwanda: a Community participatory qualitative study*’

**Table:**

Domain 1: research team and reflexivity		
**Personal characteristics**		
1. Interviewer/facilitator	Which author(s) conducted the interview or focus group?	The PI (Prof. Madeleine) led the data collection process by facilitating all Open Space Meetings, ensuring that discussions stayed focused and that all participants engaged actively. Two research assistants from the School of Nursing and Midwifery supported the process by taking detailed field notes, recording the discussions, and transcribing the audio recordings verbatim. The co-authors acted as quality controllers, reviewing the transcripts, verifying the consistency and credibility of the coding process, and providing feedback to ensure the trustworthiness and rigor of the data analysis.
2. Credentials	What were the researcher’s credentials? (e.g. PhD, MD)	The PI has PhD in community Health Nursing The two research assistant have Masters in Nursing The co-authors who played the role of “Quality controllers: Two have PhDs in Public Health (Prof Theoneste and Prof Gunilla) One has Masters in Public Health (Marie Leatitia)
3. Occupation	What was their occupation at the time of the study?	PI (Prof Madeleine) was a Post doctorate researcher fellow at the University of Gothenburg and Assoc. Professor at the University of Rwanda, School of Nursing and Midwifery Prof Theoneste Ntakirutimana was the local mentor and Professor of Public Health at the University of Rwanda and Dean of school of Public health Marie Laetitia Ishimwe Bazakare was a co-author and a Monitoring and Evaluation specialist at the University of Rwanda Prof Gunilla Kranzt was the mentor from University of Gothenburg and Senior Professor in the Department of Global Health at Gothenburg University The two research assistants (Vedaste Bagwaneza (VB) and Dieudonne Kayiranga (DK)) were the Assistant Lecturers at the University of Rwanda, School of Nursing and Midwifery
4. Gender	Was the researcher male or female?	PI is a female Local mentor (Prof Theoneste) is a male The Co-author (Marie Laetitia) is a female Prof Gunilla is a female The two research assistants (VB and DK) are males.
5. Experience and training	What experience or training did the researcher have?	The two research assistants and the PI had undertaken training in qualitative research methodologies and had previous experience of this methodology
**Relationship with participants**		
6. Relationship established	Was a relationship established prior to study commencement?	No prior relationship was established between the researchers and participants
7. Participant knowledge of the interviewer	What did the participants know about the researcher? (e.g. personal goals, reasons for doing the research)	Participants knew where the researchers worked and the purpose of the research
8. Interviewer characteristics	What characteristics were reported about the interviewer/facilitator? (e.g. bias, assumptions, reasons and interests in the research topic)	The two research assistants had an interest in the research as they have extensive experience in collecting qualitative data in communities as nurses The PI is a community health worker agent and has been working with communities for a period of time

Domain 2: study design		
**Theoretical framework**		
9. Methodological orientation and theory	What methodological orientation was stated to underpin the study? (e.g. grounded theory, discourse analysis, ethnography, phenomenology, content analysis)	The study utilized a participatory research design employing the Open Space Meeting method to collect the primary data. Eight open-space meetings were conducted in two selected districts in the northern province of Rwanda. Thematic analysis using Atlas.ti software was used to analyze the qualitative data
**Participant selection**		
10. Sampling	How were participants selected? (e.g. purposive, convenience, consecutive, snowball)	We selected participants purposively. We targeted Community Health workers, male and female parents (who have been living in the study sites for a time and who have information about the community) and Health care professionals including those working at Nutrition centers at Health centers. These participants were selected with the help of community administration.
11. Method of approach	How were participants approached? (e.g. face to face, telephone, mail, e-mail)	In the planning phase, community leaders in selected communities were contacted by the PI (Prof Madeleine) via phone calls for acceptance of the study and also their willingness to contact the participants. Upon arrival in the community on the day of data collection, the team first met with community leaders, who led us to gather participants in one of the community school rooms or one of the Sector’ office rooms. These rooms were large open areas that facilitated free movement and collaboration among participants during open-space meetings.
12. Sample size	How many participants were in the study?	A total of 194 participants participated in our open space meetings among them 109 community health workers, 80 parent’s/care givers of children under five and 5 health care professionals (nurses in charge of nutrition at the health center).
13. Non-participation	How many people refused to participate or dropped out? Reasons?	Numbers of refusals were not recorded by community leaders and once we started the data collection none dropped out. We started with 194 participants and completed our data with all 194 participants.
**Setting**		
14. Setting of data collection	Where was the data collected? (e.g. home, clinic, workplace)	The study was conducted in two districts located in the northern province of Rwanda. The Northern Province of Rwanda is known to have a high prevalence of undernutrition among children where the Northern province has been recently reported to have a stunting prevalence that was higher than the national average of 33.5% (Ngaruye et. al. 2023)^a^
15. Presence of non-participants	Was anyone else present besides the participants and researchers?	Only the researchers and participants were present
16. Description of sample	What are the important characteristics of the sample? (e.g. demographic data, date)	No other demographic information was collected about participants
**Data collection**		
17. Interview guide	Were questions, prompts, guides provided by the authors? Was it pilot tested?	Unlike traditional meetings with fixed agendas, open-space meetings allow participants to create an agenda based on topics of interest. Therefore, **the discussion topic** was “***the factors contributing to the persistence of undernutrition among under five years ‘children despite all efforts in community interventions to reduce this health issue***.” After introducing the discussion topic, the floor was given to participants to provide their views, ideas, and perceptions.
18. Repeat interviews	Were repeat interviews carried out? If yes, how many?	No repeat group sessions were required. Eight open space meetings (each one involved 20 to 25 participants) were conducted in two Districts located in the North of Rwanda.
19. Audio/visual recording	Did the research use audio or visual recording to collect the data?	The discussions were audio-recorded.
20. Field notes	Were field notes made during and/or after the interview or focus group?	Yes, Research assistants were taking filed notes.
21. Duration	What was the duration of the interviews or focus group?	The discussion duration ranged from 47 to 60 minutes. The maximum time for one meeting was one hour (60 minutes)
22. Data saturation	Was data saturation discussed?	Data saturation was not discussed as data saturation is not a core goal in the Open Space method, instead, focus is placed on capturing the breadth and diversity of contributions.
23. Transcripts returned	Were transcripts returned to participants for comment and/or correction?	Transcripts were not returned to participants

Domain 3: analysis and findings		
**Data analysis**		
24. Number of data coders	How many data coders coded the data?	Two research assistants (VB and DK) coded the data
25. Description of the coding tree	Did authors provide a description of the coding tree?	A coding Table is provided as Table 1 in the manuscript
26. Derivation of themes	Were themes identified in advance or derived from the data?	Themes were derived from the data
27. Software	What software, if applicable, was used to manage the data?	Atlas.ti
28. Participant checking	Did participants provide feedback on the findings?	No
**Reporting**		
29. Quotations presented	Were participant quotations presented to illustrate the themes/findings? Was each quotation identified? (e.g. participant number)	Quotations have been presented throughout ‘Results section’.
30. Data and findings consistent	Was there consistency between the data presented and the findings?	We reported the study findings in a clear, consistent manner in order to accurately reflect the data that have been collected Yes, major themes are clearly presented in ‘Results section’ Table 1.
31. Clarity of major themes	Were major themes clearly presented in the findings?	
32. Clarity of minor themes	Is there a description of diverse cases or discussion of minor themes?	Tale 1 indicate the themes and sub-themes.

^a^Ngaruye, I., Nzabanita, J., Niragire, F., Rizinde, T., Nkurunziza, J., Ndikubwimana, J. B., … & Ahishakiye, J. (2023). Child stunting prevalence determination at sector level in Rwanda using small area estimation. *BMC nutrition*, *9*(1), 147.
