# Supplementary material for: Factors contributing to the persistence of undernutrition among children under 5 years of age in Rwanda: a community participatory qualitative study
Source: Glob Health Action. 2025 Nov 26;18(1):2581454. doi: 10.1080/16549716.2025.2581454 (PMC12667352; doi:10.1080/16549716.2025.2581454)
Supplement: Appendix 7Completed COREQ checklist M.docx [file ZGHA_A_2581454_SM9085.docx]

**Appendix 1. Completed COREQ checklist: “***Factors contributing to the persistence of undernutrition among under five children in Rwanda: A Community participatory qualitative study”*

| **Domain 1: research team and reflexivity** | | |
| --- | --- | --- |
| **Personal characteristics** | | |
| 1. Interviewer/facilitator | Which author(s) conducted the interview or focus group? | The PI (Prof. Madeleine) led the data collection process by facilitating all Open Space Meetings, ensuring that discussions stayed focused and that all participants engaged actively. Two research assistants from the School of Nursing and Midwifery supported the process by taking detailed field notes, recording the discussions, and transcribing the audio recordings verbatim.  The co-authors acted as quality controllers, reviewing the transcripts, verifying the consistency and credibility of the coding process, and providing feedback to ensure the trustworthiness and rigor of the data analysis**.** |
| 2. Credentials | What were the researcher’s credentials? (e.g. PhD, MD) | The PI has PhD in community Health Nursing  The two research assistant have Masters in Nursing  The co-authors who played the role of “Quality controllers:  Two have PhDs in Public Health (Prof Theoneste and Prof Gunilla)  One has Masters in Public Health (Marie Leatitia) |
| 3. Occupation | What was their occupation at the time of the study? | PI (Prof Madeleine) was a Post doctorate researcher fellow at the University of Gothenburg and Assoc. Professor at the University of Rwanda, School of Nursing and Midwifery  Prof Theoneste Ntakirutimana was the local mentor and Professor of Public Health at the University of Rwanda and Dean of school of Public health  Marie Laetitia Ishimwe Bazakare was a co-author and a Monitoring and Evaluation specialist at the University of Rwanda  Prof Gunilla Kranzt was the mentor from University of Gothenburg and Senior Professor in the Department of Global Health at Gothenburg University  The two research assistants (Vedaste Bagwaneza (VB) and Dieudonne Kayiranga (DK)) were the Assistant Lecturers at the University of Rwanda, School of Nursing and Midwifery |
| 4. Gender | Was the researcher male or female? | PI is a female  Local mentor (Prof Theoneste) is a male  The Co-author (Marie Laetitia) is a female  Prof Gunilla is a female  The two research assistants (VB and DK) are males. |
| 5. Experience and training | What experience or training did the researcher have? | The two research assistants and the PI had undertaken training in qualitative research methodologies and had previous experience of this methodology |
| **Relationship with participants** | | |
| 6. Relationship established | Was a relationship established prior to study commencement? | No prior relationship was established between the researchers and participants |
| 7. Participant knowledge of the interviewer | What did the participants know about the researcher? (e.g. personal goals, reasons for doing the research) | Participants knew where the researchers worked and the purpose of the research |
| 8. Interviewer characteristics | What characteristics were reported about the interviewer/facilitator? (e.g. bias, assumptions, reasons and interests in the research topic) | The two research assistants had an interest in the research as they have extensive experience in collecting qualitative data in communities as nurses  The PI is a community health worker agent and has been working with communities for a period of time |
| **Domain 2: study design** | | |
| **Theoretical framework** | | |
| 9. Methodological orientation and theory | What methodological orientation was stated to underpin the study? (e.g. grounded theory, discourse analysis, ethnography, phenomenology, content analysis) | The study utilized a participatory research design employing the Open Space Meeting method to collect the primary data. Eight open-space meetings were conducted in two selected districts in the northern province of Rwanda. Thematic analysis using Atlas.ti software was used to analyze the qualitative data |
| **Participant selection** | | |
| 10. Sampling | How were participants selected? (e.g. purposive, convenience, consecutive, snowball) | We selected participants purposively. We targeted Community Health workers, male and female parents (who have been living in the study sites for a time and who have information about the community) and Health care professionals including those working at Nutrition centers at Health centers. These participants were selected with the help of community administration. |
| 11. Method of approach | How were participants approached? (e.g. face to face, telephone, mail, e-mail) | In the planning phase, community leaders in selected communities were contacted by the PI (Prof Madeleine) via phone calls for acceptance of the study and also their willingness to contact the participants. Upon arrival in the community on the day of data collection, the team first met with community leaders, who led us to gather participants in one of the community school rooms or one of the Sector’ office rooms. These rooms were large open areas that facilitated free movement and collaboration among participants during open-space meetings. |
| 12. Sample size | How many participants were in the study? | A total of 194 participants participated in our open space meetings among them 109 community health workers, 80 parent’s /care givers of children under five and 5 health care professionals (nurses in charge of nutrition at the health center). |
| 13. Non-participation | How many people refused to participate or dropped out? Reasons? | Numbers of refusals were not recorded by community leaders and once we started the data collection none dropped out. We started with 194 participants and completed our data with all 194 participants. |
| **Setting** | | |
| 14. Setting of data collection | Where was the data collected? (e.g. home, clinic, workplace) | The study was conducted in two districts located in the northern province of Rwanda. The Northern Province of Rwanda is known to have a high prevalence of undernutrition among children where the Northern province has been recently reported to have a stunting prevalence that was higher than the national average of 33.5% (Ngaruye et. al. 2023)^1^ |
| 15. Presence of non-participants | Was anyone else present besides the participants and researchers? | Only the researchers and participants were present |
| 16. Description of sample | What are the important characteristics of the sample? (e.g. demographic data, date) | No other demographic information was collected about participants |
| **Data collection** | | |
| 17. Interview guide | Were questions, prompts, guides provided by the authors? Was it pilot tested? | Unlike traditional meetings with fixed agendas, open-space meetings allow participants to create an agenda based on topics of interest. Therefore, **the discussion topic** was “***the factors contributing to the persistence of undernutrition among under five years ‘children despite all efforts in community interventions to reduce this health issue***.” After introducing the discussion topic, the floor was given to participants to provide their views, ideas, and perceptions. |
| 18. Repeat interviews | Were repeat interviews carried out? If yes, how many? | No repeat group sessions were required. Eight open space meetings (each one involved 20 to 25 participants) were conducted in two Districts located in the North of Rwanda. |
| 19. Audio/visual recording | Did the research use audio or visual recording to collect the data? | The discussions were audio-recorded. |
| 20. Field notes | Were field notes made during and/or after the interview or focus group? | Yes, Research assistants were taking filed notes. |
| 21. Duration | What was the duration of the interviews or focus group? | The discussion duration ranged from 47 to 60 minutes. The maximum time for one meeting was one hour (60 minutes) |
| 22. Data saturation | Was data saturation discussed? | Data saturation was not discussed as data saturation is not a core goal in the Open Space method, instead, focus is placed on capturing the breadth and diversity of contributions. |
| 23. Transcripts returned | Were transcripts returned to participants for comment and/or correction? | Transcripts were not returned to participants |
| **Domain 3: analysis and findings** | | |
| **Data analysis** | | |
| 24. Number of data coders | How many data coders coded the data? | Two research assistants (VB and DK) coded the data |
| 25. Description of the coding tree | Did authors provide a description of the coding tree? | A coding Table is provided as Table 1 in the manuscript |
| 26. Derivation of themes | Were themes identified in advance or derived from the data? | Themes were derived from the data |
| 27. Software | What software, if applicable, was used to manage the data? | Atlas.ti |
| 28. Participant checking | Did participants provide feedback on the findings? | No |
| **Reporting** | | |
| 29. Quotations presented | Were participant quotations presented to illustrate the themes/findings? Was each quotation identified? (e.g. participant number) | Quotations have been presented throughout “Results section”. |
| 30. Data and findings consistent | Was there consistency between the data presented and the findings? | We reported the study findings in a clear, consistent manner in order to accurately reflect the data that have been collected  Yes, major themes are clearly presented in “Results section” Table 1.  Tale 1 indicate the themes and sub-themes. |
| 31. Clarity of major themes | Were major themes clearly presented in the findings? |  |
| 32. Clarity of minor themes | Is there a description of diverse cases or discussion of minor themes? |  |

^1^Ngaruye, I., Nzabanita, J., Niragire, F., Rizinde, T., Nkurunziza, J., Ndikubwimana, J. B., ... & Ahishakiye, J. (2023). Child stunting prevalence determination at sector level in Rwanda using small area estimation. *BMC nutrition*, *9*(1), 147.
